# Chloroform in Labor

**Published:** 1849-06

**Authors:** 


### CHLOROFORM IN LABOR.

As the anæsthetic practice, in midwifery, is still comparatively unusual in the west, the following notice which we copy from a letter of our friend and kinsman, Dr. Wilson W. Yandell, of Gibson county, Tenn., will probably be read with interest.

"I attended, the other day, my first obstetrical case. It was a tedious case; the woman had been in labor for thirty hours before I saw her. I found the os uteri but partially dilated, hard and rounded. I administered the chloroform, and introduced the finger, as directed by Professor Miller in such cases. These means soon brought about a full dilatation, followed, in a few hours, by the birth of two fine children, a boy and a girl."

